# Dual Size-Dependent Effect of Fe_3_O_4_ Magnetic Nanoparticles Upon Interaction with Lysozyme Amyloid Fibrils: Disintegration and Adsorption

**DOI:** 10.3390/nano9010037

**Published:** 2018-12-28

**Authors:** Natália Tomašovičová, Po-Sheng Hu, Cyun-Lun Zeng, Jozefína Majorošová, Katarína Zakutanská, Peter Kopčanský

**Affiliations:** 1 Institute of Experimental Physics, Slovak Academy of Sciences, Watsonová 47, 04001 Košice, Slovakia; nhudak@saske.sk (N.T.); majorosova@saske.sk (J.M.); zakutanska@saske.sk (K.Z.); kopcan@saske.sk (P.K.); 2 Institute of Photonic System, National Chiao Tung University, Tainan City 71150, Taiwan; 3 Institute of Imaging and Biomedical Photonics, National Chiao Tung University, Tainan 71150, Taiwan; jean4381661@gmail.com

**Keywords:** magnetic nanoparticles, colloidal suspension, amyloid fibrils

## Abstract

Nanomedicine compounds containing nanoparticles, such as iron oxides and gold, have been demonstrated to be effective in promoting different magnitudes of interaction with amyloid β fibrils, of which disintegrating or inhibiting effects are of great importance to treating fibrillary aggregation-induced neurological disorders such as Alzheimer’s disease. This research herein studies the interaction between lysozyme amyloid fibrils, a type of fibers derived from hen egg white lysozyme, and Fe_3_O_4_ magnetic nanoparticles (MNPs) of an assorted diameter sizes of 5 nm, 10 nm and 20 nm, using atomic force microscopy (AFM). Specifically, the effects of the sizes of negatively charged MNPs on the resultant amyloid fibrillary mixture was investigated. Our results of AFM images indicated that the interaction between MNPs and the fibrils commences immediately after adding MNPs to the fibril solution, and the actions of such MNPs-doped fibrillary interplay, either integration or segmentation, is strongly dependent on the size and volume concentration of MNPs. In the cases of 5 nm and 20 nm particles of equivalent volume concentration, the adsorption and agglomeration of MNPs onto the fibrillary surfaces was observed, whereas, interestingly, MNPs with diameter size of 10 nm enables segmentation of the slender fibrils into debris when a proper implemented volume concentration was found, which signifies utter destruction of the amyloid fibrillary structure.

## 1. Introduction

Deposition of a variety of misfolded proteins of amyloidal form in living organism induces detrimental cytotoxicity that lethally disrupts functions of cell-specific organs [1], consequentially resulting in a number of diseases ranging from Alzheimer’s [2] and Parkinson’s diseases [3] to diabetes type II [4]. Particularly with neurological disorders, memory loss and nerve trembling, the respective symptoms of Alzheimer’s and Huntington’s diseases, are the results of neuronal degradation caused by the aggregates of amyloid β-peptides together with other aggregating groups of genetically abnormal tau protein and FTDP-17 [5]. These diseases inflict great mishaps on patients’ lives, and thus spurs numerous development of therapeutic tools, such as nanomaterial systems, to disintegrate or inhibit the disease-causing fibrillary assembly. Nanoparticles (NPs), owing to their unique physical and biochemical properties, have drawn much interests to experimentally treating a handful of neurological diseases [6,7,8], and one example of the usefulness of NPs is to trace the growth of β-lactoglobulin amyloid fibrils [9]. To gain more insight into interaction between amyloid fibrils and NPs, previous research studies reported by Majorosova et al. and Chen et al. respectively, revealed that the binding adsorption of magnetite nanoparticles onto the surfaces of lysozyme and insulin amyloid fibrils are attributed to the van der Waals forces since both fibrils and NPs have positive zeta potential [10,11]. In addition, regardless of ionic strength, pH value of the mixture solution of lysozyme proteins and silica NPs was found influential on the degree of structural agglomeration [12]. Up until now, a handful of bared NPs such as gold (Au), iron oxides (Fe_3_O_4_), tungsten sulfide (WS_2_), zinc oxide (ZnO), graphene oxide and etc. have already been demonstrated with positive effects of inhibition or dissociation upon the amyloid β aggregation of different protein origins [13,14,15,16,17]. Besides random fibrillary interaction with bare NPs, many aspects of NPs such as, just to name a few, sizes, shapes, surface charges and pH of solution, and hydrophobicity, have already been exploited to interrogate the feasibility of reversing or destroying the tendency of fibrillary assembly [18,19]. From the perspective of surface charge of NPs, for instance, although individual protein monomers herald negative electric charge, the assembly of the monomers transform the zeta potential of molecular complex into positive, which serves as a standard assay to screening types of NPs that can be used to prevent the formation of fibrillary structures as well as to disintegrate the matured amyloid fibrils. Liao et al. confirmed that negatively charged bare AuNPs can effectively dissociate amyloid β-40 fibrils or prevent the formation of fibrillary structure resulting in mitigation of neurotoxicity [20]. Likewise, Fe_3_O_4_ magnetic fluids coated with different amount of bovine serum albumin that regulates hydrodynamic diameter, zeta potential and isoelectric point has been verified with its ability to depolymerize the complex of lysozyme amyloid fibrils [21], and similar concentration-dependent results carried out with glutathione-wrapped and curcumin-functionalized AuNPs were also reported [13,22]. To make further elucidation into whether the surface charge of NPs indeed plays a critical role for fibrillary disintegration, a comprehensive study on the effects of electric polarity of NPs on amyloid β fibrils with segments 1–40 was carried out by coating AuNPs with citrate and cetyltrimethylammonium bromide, rendering negatively and positively charged complex, respectively, which was observed using an amyloid protein-binding dye, thioflavin T (ThT) and signifies destruction of fibrillary structure for as long as the molecular complex is electrically negative [23]. Although the results of numerous studies on amyloid fibrillary aggregation were presented by the regulation of ThT fluorescence, it was also demonstrated that ThT has the destructive ability upon features of metastable protein mutants, reducing the toxicity induced by amyloid β aggregates [24]. Moreover, despite numerous research have been done on interaction between amyloid fibrils and NPs inherited with different physical and biochemical characteristics, a systematic study on how the sizes of NPs influence the fibrillary aggregates is still lacking. One exception was a particle size-dependent experiment where authors described different morphology of amyloid β fibrils formed on a lipid bilayer extracted from brain when the protein monomers are incubated with different sizes of AuNPs, but without commenting any inhibitive or disintegrating effects [25]. This research herein is poised to present, comprehensively, the dependence of amyloidal fibrillary aggregation on the sizes of negatively charged NPs. Experimentally, by ruling out the factors of the polarity of surface charges and unnecessary influence of fluorescent dyes, the self-assembled bio-inorganic composite composed of lysozyme amyloid fibrils doped with carboxyl iron oxide (Fe_3_O_4_) NPs of different sizes, 5 nm, 10 nm and 20 nm and of a variety of volume concentrations are investigated using atomic force microscopy (AFM). The AFM images are processed and analyzed using NanoScope software which is freely accessible.

## 2. Materials and Methods

### 2.1. Sample Preparation

**Figure 1:**
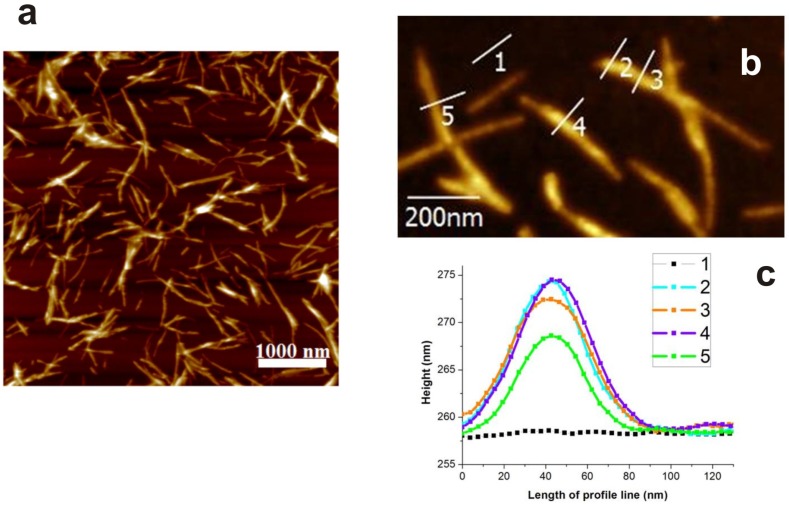
AFM images of (**a**) pure LAF and (**b**) a close-up view of the same sample in (**a**), and (**c**) topographical profiles of fibrillary thickness indicated in (**b**).

Hen egg white lysozyme (HEWL) (lyophilized powder, lot number L6876, 50,000 units per mg protein) was obtained from Sigma-Aldrich Chemical Company (St Louis, MO, USA). All other chemicals were purchased from either Sigma or Fluka and were of analytical reagent grade. The protocol of preparing the solution of lysozyme amyloid fibrils (LAFs) and NP-incorporated fibrillary assembly is of the following. In brief, HEWL powder was dissolved in 0.2 M glycine-HCl with pH of 2.2 and 80 mM NaCl to obtain a final concentration of 5 mg/mL. The prepared solution was heated for 2 h at 65 °C in an enclosed bottle with constant stirring at the speed of 250 round per minutes (rpm), of which the final appearance delineated by AFM imaging is shown in Figure 1.

**Figure 2:**
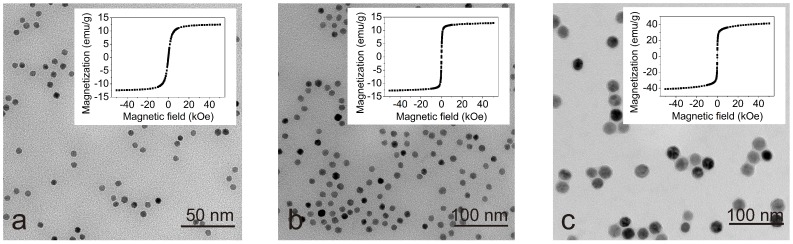
TEM images of (**a**) 5 nm, (**b**) 10 nm and (**c**) 20 nm MNPs as well as the respective magnetization curves shown in the inset plots are presented.

The spherical carboxyl iron oxide NPs of an assorted sizes of 5 nm, 10 nm and 20 nm dispersed in deionized (DI) water (H_2_O) with Fe concentration of 5 mg/mL containing 0.02% of NaN_3_ were purchased from Ocean Nano Tech Co. (San Diego, CA, USA). The NPs were coated with a monolayer of amphiphilic polymer atop a monolayer of oleic acid, which are used for conjugation with the surface of biological elements and dispersion in the acqueous solution, and retention of the particle shape, respectively [26,27]. The size and magnetic properties were examined by transmission electron microscopy (Philips CM120) and by SQUID magnetometer (Quantum Design MPMS 5XL), respectively, which are presented in Figure 2. As can be seen from the figure, particles of all sizes are well-dispersed, no agregates were observed. The magnetic NPs exhibit superparamagnetic behavior and the saturation magnetization increases as the nanoparticle size enlarges, which is attributed to the size of the particles as well as their atomic structure and fraction of atoms on the surface of the particles [28,29].

These MNPs are incorporated with the LAF solution after the completion of the fibrillation process.

### 2.2. Atomic Force Microscopy

AFM is utilized in this research for its providence of topographical visualization of the interaction between LAF and NPs without complicating the detailed biochemical and structural properties at molecular level. The prepared samples including pure LAF as well as LAF doped with magnetic nanoparticles were casted on the surface of freshly cleaved mica, adsorbed for 5–10 min, and removed with the redundant sample by rinsing dropwise with ultrapure water. The drying of the samples as well as all measurements were carried out in an aerated ambient temperature environment with humidity in the range of 30–40% and the nominal ambient temperature is 26 °C The samples-loaded mica slide was then placed on the sample platform for image acquisition using the tapping-mode of atomic force microscopy (Veeco di Innova, Bruker AXS Inc., Madison, WI, USA) in air environment, and the AFM tip used throughout the entire experimental session was the antimony-doped etched silicon probe with aluminum thin film coated on its backside (Bruker, Billerica, MA, USA).

### 2.3. Zeta Potential

The values of zeta-potentials for individual samples were measured using Zetasizer (Nano ZS, Malvern Instruments, Herrenberg, Germany). The zeta-potential of MNPs solution was negative and of fibril solution was positive, which are summarized in Table 1. Also, all fibrillary composites incorporated with MNPs have positive zeta-potential slightly varied between 40 mV and 48 mV.

## 3. Results and Discussion

In our previous work [30], the interaction between spherical as well as spindle particles with LAF indicated that spherical MNPs attach well to the surface of LAF, which is in strong agreement with the results associated with fibrils of different protein origin like β-lactoglobulin, insulin and lysozyme and NPs such as iron oxide, Ag and Au [11,25,31,32,33,34], whereas the spindle-like MNPs did not promote any significant interaction with LAF. From the perspectives of the particle size, while spherical magnetic particles with hydrodynamic diameter of 26 nm promotes strong affinity with LAF, the spindle-like magnetic nanoparticles with the length of 203 nm have rather a limited interaction with the LAF, which leads to our hypothesis that the sizes of MNPs may play an important role in various extent of interaction including the disintegration of and aggregation with LAF.

**Figure 3:**
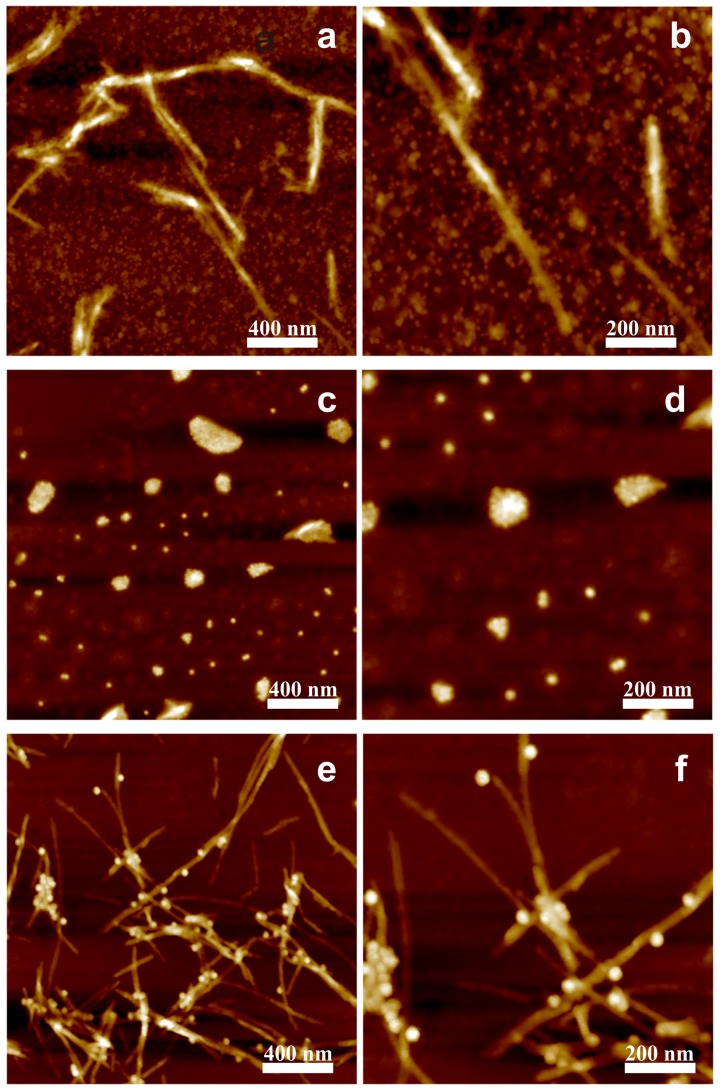
AFM images of LAF doped with MNPs of diameter sizes of (**a**,**b**) 5 nm, (**c**,**d**) 10 nm, and (**e**,**f**) 20 nm. All right-hand panels are magnification of that on the left.

Thereafter, to testify our hypothetical surmise that size or shape may play an important role in such a non-interactive scenario, the dependence of the fibrillary assembly on the sizes of negatively charged spherical iron oxide MNPs, 5 nm, 10 nm and 20 nm was investigated systematically. A fixed amount of LAF of 300 μL was incorporated with 30 μL of the MNPs of different sizes to elucidate the resultant interaction. The concentration of LAF was fixed because the preliminary experiments showed that this concentration of LAF is stable in time and this way some sedimentation process in LAF solution will not influence the interaction. Figure 3 illustrates AFM images of the topographical surface of the bio-inorganic fibrillary composites. As can be seen from the Figure 3a,b, some 5 nm NPs are adsorbed onto the surface of the fibrils while many remain floating in the dried fibril bath. Additionally, when the fibrillary assembly is incorporated with 20 nm NPs, a pronounced agglomeration of NPs onto the fibrillary surface can be observed in Figure 3e,f, however, without any remnant floating NPs. This disparity in the number of free NPs is attributed to the fact that the number of 5 nm NPs is more than 60 times higher than that of 20 nm NPs at the equivalent volume concentration; on the side of similarity, the slender appearance of the fibrils, as presented in Figure 1, of both cases remain intact.

**Figure 4:**
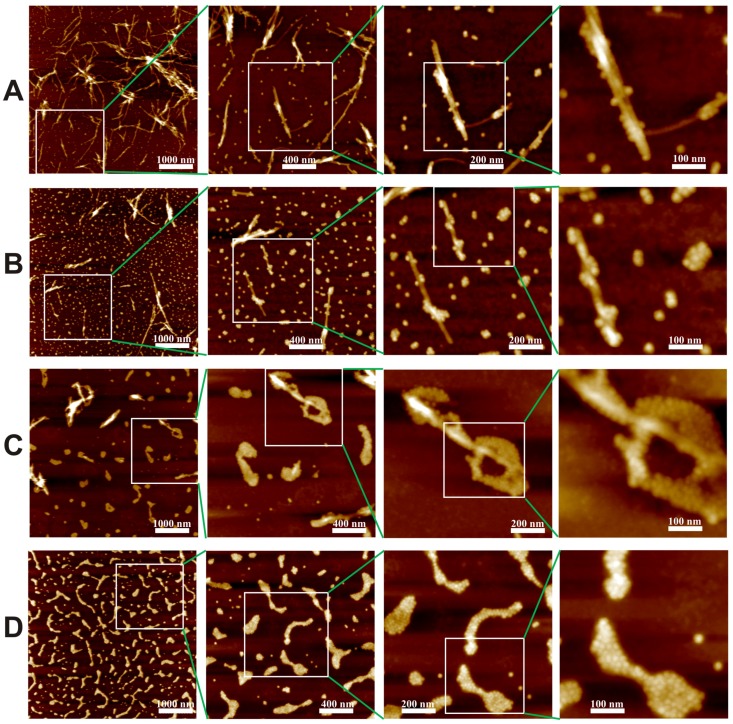
AFM images of the resultant self-assembled LAF incorporated with an array of volume concentration of 10 nm NPs, (**A**) 10 μL, (**B**) 15 μL, (**C**) 20 μL and (**D**) 30 μL at increasing scales of resolution from left to right is illustrated, and each zoomed-in is enclosed by a white rectangle from the preceding image of lower magnification.

**Figure 5:**
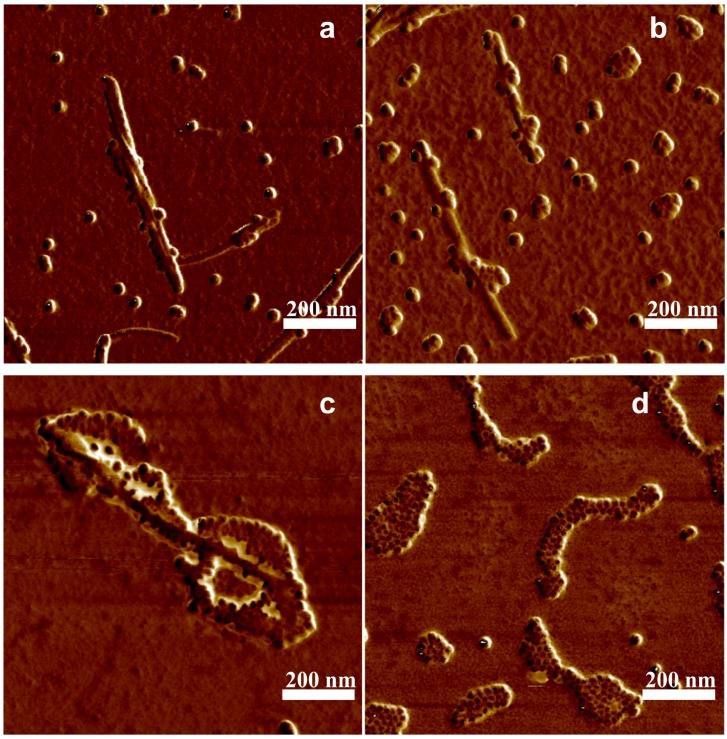
AFM phase image of lysozyme fibrils doped with magnetic nanoparticles with diameter of 10 nm. The volume of (**a**) 10 μL, (**b**) 15 μL, (**c**) 20 μL, (**d**) 30 μL of MNPs solution was added to 300 μL of LAF5.

Contrary to the adsorptive results of 5 nm and 20 nm NPs, only the clusters of 10 nm NPs can be found in Figure 3c,d. The experiment was repeated three times for a solid confirmation that 10 nm NPs may be heralded with disintegrating effects upon LAF. Indeed, the presented results illustrate that the effective fibrillary segmentation by 10 nm MNPs is attributed to the particle size which is in close proximity to the fibrillary dimension, namely, the height of the fibrils shown in Figure 1. To further reveal how 10 nm NPs influence the self-assembly of LAF, the samples of lower volume concentration, 10 μL, 15 μL, 20 μL, were implemented. Figure 4A–D depicts the resultant fibrillary structure doped with respective volume concentration at four different scales of resolution. In the cases of the NPs solution of 10 μL, the corresponding resultant structures resemble those infused with 5 nm and 20 nm NPs, and 15 μL produces similar structures accompanied with slightly larger free clusters of NPs. As the concentration raises up to 20 μL, the fibrillary debris with irregular shapes covered or surrounded by NPs as well as the free floating NPs clusters can be observed from Figure 4C. Similarly, the results of 30 μL produces even higher density of the debris of fragmented fibrils covered with 10 nm NPs alongside random distribution of NP clusters, which exhibit blurred granular-like appearance in the image of highest magnification. To overcome such aberration, the phase domain of AFM images of all concentration are depicted and shown in Figure 5 which clarifies the distribution of 10 nm NPs clusters and elucidates resultant morphologies of the bio-inorganic interaction, suggesting destructive fragmentation of the fibrils.

**Figure 6:**
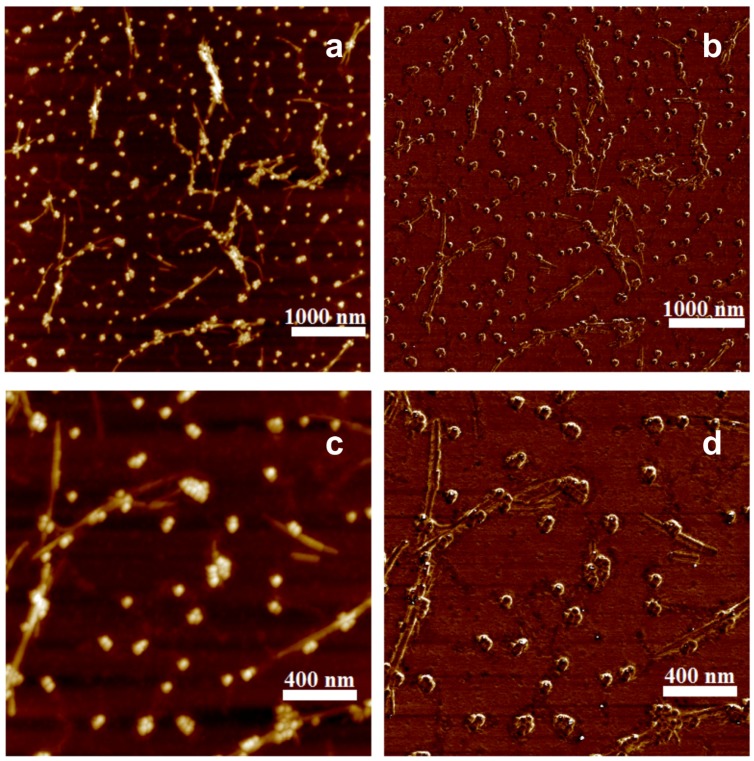
AFM images of LAF doped with 300 μL of MNPs of 20 nm diameter size present topographical measurement in (**a**,**c**) surface height and (**b**,**d**) phase domain of the sample, where the lower panels are the respective magnification of the upper ones.

Also notice in Figure 2 that the superparamagnetic behavior was confirmed for the MNPs of all sizes and the saturation magnetization increases as the particle size enlarges. However, in the case of present experiment the magnetization is not a pertinent material property because although 10 nm particles have the same magnetic properties in all experimental cases, but when its concentration is lower than some critical level, their fibrillary interaction behave similarly to that of smaller and larger particles. Moreover, in our experiment, all particles were covered with the same negatively charged polymer coating and this way we do not suppose the coating as well as the surface charge plays an important role in the NP-incorporated fibrillary interaction. Likewise, the polarity of zeta potential of NP solution of all sizes and the pure LAF presented in Table 1 indicate the mutually attractive electrostatic forces the main interactive mechanism between the NPs and the fibrils. The interaction of 10 nm particles differs from that of 5 nm and 20 nm particles suggests that the observed effect, as indicated by our results, is connected with size of particles, and implies that surfactant as well as zeta potential of nanoparticles are not the major factors for this interaction. Atop the study of volume concentration-dependent fibrillary interaction, 300 μL of 20 nm NP solution is employed to examine whether larger amount of the MNPs allows similar interaction upon the fibrillary structure, to verify our findings associated with low volume concentration that only the concentration of appropriate size, 10 nm in the present study, can render destructive impact on the fibrils. Figure 6 presents topographical and phase-domain AFM images on left (a,c) and right panels (b,d), respectively. As clearly indicated in the figure, the NPs solution of higher volume concentration promotes to some extent the NP adsorption on the fibrillary surface without gross accumulation of NP considering the number and size of the NPs, and also many free floating NPs and NP clusters can still be found across the entire images. This subset experiment confirmed that at very large concentration of 20 nm particles no destruction of the amyloid fibrils, as opposed to the case of 10 nm particles where the quantity of particles is larger because at the same volume concentration of nanoparticles the number of particles dramatically increases with incremental decrease in size, was observed. Furthermore, concerning the treatment of alzheimer’s disease or alike, previous studies suggested that acidification of brain is one of the cerebral signature that indicates pathogenesis of neurological diseases [35,36], with pH value ranging from 5 to 6. Regarding neurodegenerative disease, although the range of environmental pH for alzheimer’s disease is not specified, Prasad et al. [36] demonstrated that the lowering of anionic exchanger, Na^+^ and H^+^ in astrocytes can lead to acidification of endosome, which results in defective amyloid beta (Aβ) peptide. In the research herein, however, to synthesize the lysozyme amyloid fibrils, the experimental fibrillary model, from hen egg white in the form of lyophilized powder, within a few hours, the dissolving of the protein powder necessitates the utilization of acid such as glycine-HCl, rendering fibrillary solution with an overall pH value of 2.2, which is lower than the pH value of neurological diseases. Therefore, treatment of the disease from the perspective of environmental pH, requires additional detailed experiment to clarify whether change of pH value in the environment still promotes the similar interaction observed in this study. Also, further application of the 10 nm MNPs upon neurofibrils like human amyloid beta 42 is necessitated to confirm its disintegrating function in treating amyloidal protein aggregation-induced neurological disorders. In summary, the results presented in this research corroborate that the size as well as concentration of MNPs can play adsorbing and segmenting roles in the self-assembly of LAF.

## 4. Conclusions

Research presented in this paper demonstrates dual effects of negatively charged carboxyl-group-attached Fe_3_O_4_ NPs, adsorption and disintegration, upon LAF, and such effects are highly dependent on particle sizes and volume concentration. In essence, the 30 μL of 10 nm NP solution utterly disintegrates the LAF into fragmented debris, which may be applicable to treating fibrillary aggregation-induced neurodegenerative diseases, and other volume concentrations promote limited adsorption or partial destruction onto the fibrils; and NP solution of all other particle sizes allow different degree of adsorbed aggregation may open up a new avenue for the novel design of functional bio-inorganic devices. Lastly, this study not only clarifies obscurity of general findings brought by many previous studies on fibrillary assembly doped with nanomaterials harnessed with a variety of physical, and electrical properties, but also create unprecedented opportunity for other applications. The results presented in this research corroborate that the size as well as concentration of MNPs can play adsorbing and segmenting roles in the self-assembly of lysozyme amyloid fibrils, which may provide an alternative method to treating fibrillary aggregation-induced neurological disorders mentioned earlier, and enables the design of novel devices with unprecedented functions using optical, electrical and magnetic manipulation.

## Figures and Tables

**Table 1:** Zeta potential of magnetic particles and pure LAF solution.

**Sample**	5 nm MNPs	10 nm MNPs	20 nm MNPs	LAF
**Zeta potential**	−58.9 mV	−55.4 mV	−31.5 mV	44.8 mV

## Abbreviations

The following abbreviations are used in this manuscript:

AFM	Atomic force microscopy
LAF	Lysozyme amyloid fibrils
HEWL	Hen egg white lysozyme
MNP	Magnetic nanoparticle
NP	nanoparticle
TEM	Transmission electron microscopy
